# *Bordetella bronchiseptica* infections in patients with HIV/AIDS: A case report and review of the literature

**DOI:** 10.1097/MD.0000000000028244

**Published:** 2021-12-23

**Authors:** Veena R. Gujju, Bushra Akram, Dena R. Shibib, Miranda A. McGhee, Douglas A. Drevets

**Affiliations:** a Department of Medicine, University of Oklahoma Health Sciences Center, Oklahoma City, OK; b Department of Pathology and Laboratory Medicine, University of Oklahoma Health Sciences Center, Oklahoma City, OK; c Department of Pathology and Laboratory Medicine, Oklahoma City VA Health Care System, Oklahoma City, OK; d Department of Medicine, Section of Infectious Diseases, University of Oklahoma Health Sciences Center, Oklahoma City, OK.

**Keywords:** *Bordetella bronchiseptica*, human immunodeficiency virus/acquired immunodeficiency syndrome, pneumonia, respiratory pathogens, zoonotic infections

## Abstract

**Rationale:**

*Bordetella bronchiseptica* is a common cause of upper respiratory tract infections in domesticated dogs and cats and a rare zoonotic pathogen in immunocompromised humans. With increasing numbers of people acquiring pets and spending time with them in confined spaces due to COVID-19 lockdowns, it is important to be aware of adverse health consequences brought about by this interaction. We present a case of *B bronchiseptica* pneumonia in a patient with human immunodeficiency virus/acquired immunodeficiency syndrome (HIV/AIDS) and review key characteristics of an additional 30 cases of *B bronchiseptica* infections in 29 patients with HIV/AIDS that were identified by literature review.

**Patient concerns:**

A 61-year-old male with HIV/AIDS who was not on antiretroviral therapy and had advanced immunosuppression with a CD4+ T-lymphocyte count of 3 cells/μL sought medical attention for multiple somatic issues including subjective fevers, shortness of breath, and intermittent chest pain.

**Diagnosis:**

Computed tomography of the chest identified bilateral nodular opacities in the lower lobes with scattered areas of ground glass opacities. *B bronchiseptica* was identified in sputum culture by mass spectrometry followed by supplementary biochemical testing.

**Interventions:**

Empiric broad-spectrum antibiotics were initiated and changed to levofloxacin after susceptibility testing was completed.

**Outcomes:**

The patient was discharged after symptomatic improvement with levofloxacin.

**Lessons:**

Pneumonia with interstitial infiltrates in the setting of advanced CD4 lymphocyte depletion is the most common clinical syndrome caused by *B bronchiseptica* in patients with HIV/AIDS, and may be accompanied by sepsis. Advanced immune suppression, as well as chronic medical conditions, for example, alcoholism, diabetes, and renal failure that compromise host defenses are also commonly found in cases of *B bronchiseptica* infection in patients who do not have HIV infection. Reported animal contact among patients was not universal. Isolates were susceptible to aminoglycosides, carbapenems, fluoroquinolones, but typically resistant to most cephalosporins.

## 1 Introduction

*Bordetella bronchiseptica* is a gram negative coccobacillus that frequently causes respiratory tract infections in domesticated mammals and is a rare cause of zoonotic infections in humans. It is most commonly associated with canine infectious tracheobronchitis known as “kennel cough”, and contributes to atrophic rhinitis in swine.^[1–3]^ In these animals manifestations range from asymptomatic carriage to pneumonia with ensuing death. Domestic cats are also susceptible to *B bronchiseptica*, particularly those in crowded environments or multi-cat households.^[1,2,4]^ Key bacterial virulence factors include adhesions required for tracheal colonization, autotransporters, and a type 3 secretion system to inject toxins including adenylate cyclase, dermonecrotic toxin, and tracheal cytotoxin into host cells.^[2,5,6]^*B bronchiseptica* can invade and survive within phagocytic and nonphagocytic cells and cell lines, including tracheal epithelial cells, but lacks the ability to replicate intracellularly.^[7]^ Host defenses against *B bronchiseptica* depend on IFN-γ stimulation of host cells and antibodies.^[8–10]^

*B bronchiseptica* is shed in nasal and respiratory secretions of infected animals.^[1,2]^ Thus, spread to humans occurs through direct contact with infected respiratory secretions and presumably also by aerosolized respiratory droplets as shown for *Bordetella pertussis*.^[3,11]^ Human infection by *B bronchiseptica* is rare with only 5 cases cited in a 1980 review, 25 in 1991, and approximately 90 cases reported in 2009.^[1,3,12]^ Data suggest that most humans infected by *B bronchiseptica* are immunocompromised, with the initial reports of infections in patients with human immunodeficiency virus/acquired immunodeficiency syndrome (HIV/AIDS) occurring in 1991.^[3,13]^ Interestingly, *B bronchiseptica* is not included among diseases that can spread between humans and animals on the Centers for Disease Control and Prevention website (https://www.cdc.gov/healthypets/diseases/index.html). Nonetheless, the SARS-2-CoV pandemic spurred an increase in pet adoption suggesting that higher numbers of vulnerable people could come into contact with this bacterium.^[14]^ Here, we describe a case of *B bronchiseptica* in a patient with HIV/AIDS and reviewed the literature for similar cases to identify key epidemiologic, clinical, radiographic, and microbiological features of this unusual zoonosis. Such a series has not been reported in the past 20 years despite changes in diagnostic techniques and antimicrobial usage. These results will be useful for guiding clinicians who care for immunocompromised patients.

## 2 Case report

**Figure 1:**
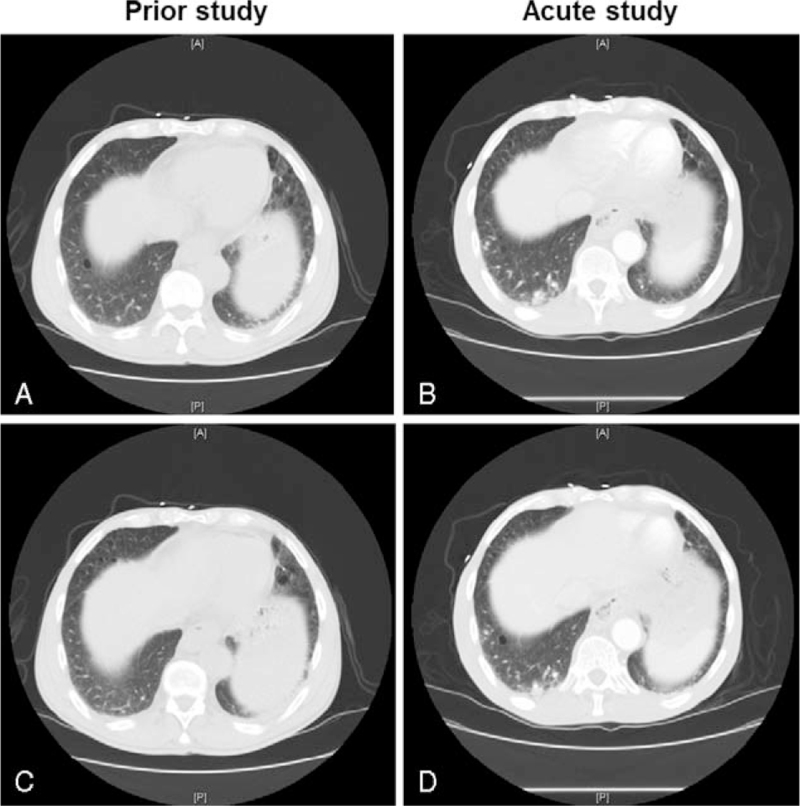
Chest imaging of *B bronchiseptica* pneumonia. Computed tomographic slices show similar levels (A & B, C & D) from a study obtained one year prior to the acute illness (A, C) compared with the study during the acute illness with *B bronchiseptica* (B, D). Images show nodular opacities in the right lower lobe.

A 61-year-old male diagnosed with HIV for greater than 15 years with inconsistent antiretroviral use sought medical attention for subjective fevers, shortness of breath, intermittent chest pain, odynophagia, and weight loss. On presentation, the patient was afebrile, vitally stable, with an oxygen saturation >94% on room air. Physical exam revealed normal respiratory sounds and oral candidiasis. The WBC was 1130 cells/μL (reference: 4000–10,000 cells/μL) with an absolute neutrophil count of 620 cells/μL (reference: 1600–8600 cells/μL). The CD4 count was 3 cells/μL (reference: 200–3390 cells/μL) and the HIV RNA was 109,000 copies/mL (reference: not detected) Computed tomography of the chest revealed nodular opacities in both lower lobes with scattered areas of ground glass opacities consistent with pneumonia (Fig. 1). The patient was admitted and started on empiric, broad-spectrum antibiotics including trimethoprim/sulfamethoxazole (TMP/SMX). Gram stain of induced sputum showed 2 to 5 WBCs/field with moderate Gram positive rods, few Gram positive cocci in clusters, and few Gram negative rods. Culture yielded normal oral flora along with *Staphylococcus aureus* and small white colonies of Gram negative rods on sheep blood agar. Identification by matrix assisted laser desorption ionization time of flight (Bruker, Billerica, MA) mass spectrometer resulted as *Bordetella* group to include *B pertussis/B bronchiseptica/Bordetella parapertussis* (log score 2.66). Supplementary testing differentiated between the 3 species; the isolate was motile and positive for both oxidase and urease which led to identification as *B bronchiseptica*. *B pertussis*, and *B parapertussis* are both nonmotile; *B parapertussis* is oxidase negative and *B pertussis* is urease negative.^[15]^ After susceptibility testing (Trek Sensititre, ThermoFisher Scientific, Waltham, MA), antibiotics were changed to levofloxacin 500 mg daily and the patient was discharged home after improvement to complete 14 days of antibiotics. Notably, the patient denied recent animal exposure.

After discharge blood cultures turned positive for *Mycobacterium avium*. However, the patient did not keep follow up appointments in the Infectious Diseases clinic and did not return phone calls post-discharge or during the writing of this report. Informed consent was waived as the Institutional Review Board (IRB) of the Office of Human Research Participant Protection at the University of Oklahoma Health Sciences Center, Oklahoma City, OK was consulted and determined that this case report did not meet the definition of human subject's research.

## 3 Results of literature review

A Medline search was done to identify cases of *B bronchiseptica* in patients with HIV/AIDS. Search terms included “*Bordetella bronchiseptica*”, “HIV”, and “AIDS”. Articles in languages other than English were excluded. The search identified an additional 30 cases in 29 patients since 1991, the characteristics of which are shown in Table 1. Most cases were reported in the 1990s (19/31, 61%) with a median age of 35 (range 21–61) and 73% (22/30) were male. CD4 lymphocyte counts were reported for 24/30 (80%) patients showing a median of 32.5 CD4 cells/μL (range 0–212) with only 2 cases in patients with CD4 counts ≥ 200 cells/μL. Animal exposure history was given for 73% (22/30) of patients with 55% (12/22) patients reporting animal contact. Of these, 7/12 (58%) reported contact with dogs and 3/12 (25%) reported contacts with only cats. One patient reported contact with both cats and dogs, and 1 reported contact with multiple species as he was a kennel worker.

**Table 1:** Case characteristics.

Year of report	Age	Sex	# CD4 (cells/μL)	Animal exposure	Clinical syndrome(s)	Culture source	Definitive treatment	Outcome	Citation
1991	61	Male	200	No	Pneumonia	BAL	Ciprofloxacin	Death	Amador et al
1991	38	Male	n/a	New puppy	Pneumonia	BAL	Ceftazidime ciprofloxacin	Recovered	Decker et al
1992	43	Male	n/a	n/a	Pneumonia	BAL	Erythromycin ciprofloxacin	Recovered, persistent symptoms	Ng et al
1992	33	Male	n/a	Cat	Bacteremia	Blood	Imipenem	Recovered	Qureshi et al
1993	32	Female	56	No	Pneumonia	Sputum	Amoxacillin	n/a	Mesnard et al
1994	28	Male	73	Dogs cats	Pneumonia	Sputum	Ciprofloxacin	Recovered	de la Fuente et al
1995	29	Male	46	No	Pneumonia	Sputum	Clarithromycin	Recovered	Libanore et al
1995	28	Male	n/a	Kennel worker	Pneumonia	Sputum	Ceftazidime	Recovered	Woodard et al
1998	33	Female	100	no	Pneumonia	Sputum, BAL	Tobramycin doxycline	Recovered	García San Miguel et al
1999	28	Male	7	n/a	Sepsis	Bone marrow	n/a	n/a	Dworkin et al
	24	Male	n/a	n/a	Pneumonia	Sputum, BAL	n/a	n/a	
	37	Male	22	n/a	Pneumonia	BAL	n/a	n/a	
	28	Female	35	n/a	Sinusitis	Nasal	n/a	n/a	
	26	Female	7	Cats	Sinusitis	Nasal	n/a	n/a	
	34	Male	10	n/a	Sinusitis, pneumonia	BAL	n/a	n/a	
	33	Male	8	Dog	Bronchitis	Sputum	n/a	n/a	
	36	Female	1	No	Pneumonia	BAL	n/a	n/a	
			2	No	Bronchitis	Sputum	n/a	n/a	
	35	Male	153	Dog	Pneumonia	BAL	n/a	n/a	
2002	34	Female	25	Dog	Pneumonia	Sputum, BAL	Ofloxacin	Recovered	Lorenzo-Pajuelo et al
	26	Male	97	No	Pneumonia	Sputum, BAL	Vancomycin rifampin ciprofloxacin	Recovered	
2002	38	Male	39	No	Pleural effusion, pneumonia	Pleural fluid	Ceftazidime	Recovered	Viejo et al
2009	44	Male	93	Cat	Pneumonia, bacteremia	Blood, BAL	Ciprofloxacin gentamicin	Recovered	Mazumder et al
2009	42	Male	20	Dog	Pneumonia	Sputum	Levofloxacin	Recovered	Galeziok et al
2011	42	Female	n/a	n/a	Pneumonia	BAL	TMP/SMX	Recovered	Wernli et al
	35	Male	n/a	n/a	Bronchitis	BAL	n/a	Recovered	
2016	43	Male	212	n/a	Pneumonia	Sputum	Levofloxacin	Recovered	Rampelotto et al
	49	Male	60	Dogs	Pneumonia, sepsis	Sputum	Ciprofloxacin, meropenem	Death	
2019	24	Male	0	Dog	Pneumonia, sepsis	BAL	Moxifloxacin	Recovered	Sameed et al
2019	52	Female	30	No	Pneumonia	BAL	Levofloxacin	Recovered	Gupta et al
2021	61	Male	7	No	Pneumonia	Sputum	Levofloxacin	Recovered	This report

n/a = value not available or not reported.

Nearly all clinical episodes (30/31, 97%) involved respiratory tract infections, the most common being pneumonia (24/31, 77%) (Table 1). Less common syndromes included sinusitis (3/31, 10%), bronchitis (2/31, 6%), bacteremia (2/31, 6%), and pleural effusion (1/31, 3%), often accompanied by pneumonia. Table 2 details radiographic findings and shows interstitial infiltrates alone are the most common finding and less frequently appear with consolidation or cavitation. Outcomes data was available for 20 of 31 episodes and shows a case fatality rate of 10% (2/20) (Table 1). One patient was reported as recovered but with persistent symptoms.

**Table 2:** Antibiotic susceptibility table.

Antibiotic			
Class	Drug	Isolates tested (n)	Susceptible (%)
Aminoglycosides	Amikacin	9	100.00
	Gentamicin	8	100.00
	Tobramycin	10	90.00
Carbapenams	Imipenem	6	100.00
	Meropenem	2	100.00
Cephalosporins (generation)	Cefazolin (1^st^)	7	0.00
	Cephalotin (1^st^)	6	0.00
	Cefuroxime (2^nd^)	8	0.00
	Cefotaxime (3^rd^)	2	0.00
	Ceftazidime (3^rd^)	11	63.64
	Ceftriaxone (3^rd^)	7	0.00
	Cefepime (4^th^)	3	0.00
Fluoroquinolones	Ciprofloxacin	9	100.00
	Levofloxacin	2	100.00
	Ofloxacin	4	100.00
Monobactams	Aztreonam	7	14.29
Penicillin's +/- beta-lactamase inhibitors	Amoxicillin/clavulanic acid	4	100.00
	Ampicillin	13	23.08
	Ampicillin/sulbactam	2	0.00
	Piperacillin/tazobactam	6	83.33
	Ticarcillin/clavulanic acid	5	100.00
Polymyxins	Colistin	2	100.00
Antifolates	Trimethoprim-sulfamethoxazole	15	100.00
Tetracyclines	Tetracycline	4	75.00

*B bronchiseptica* was most commonly isolated from respiratory secretions including bronchoalveolar lavage specimens (16/31, 52%), and sputum (14/31, 45%) (Table 1). It was occasionally cultured from blood (2/31, 7%), bone marrow (1/31, 3%), and pleural fluid (1/31, 3%). Results of antimicrobial susceptibility testing are reported in Table 3. Carbapenems and fluoroquinolones were uniformly active. Aminoglycosides and various beta-lactam/beta-lactamase inhibitor combinations in 15/18 (83%) were also highly active. In contrast, TMP/SMX was active against 8/16 (50%) of isolates and first and 2^nd^ generation cephalosporins were not active against any isolates whereas of the 3^rd^ and 4^th^ generation cephalosporins, only ceftazidine showed activity (7/12, 58%). These results are similar to a compilation of antimicrobial susceptibility testing results showing reliable activity of aminoglycosides, imipenem, ciprofloxacin, and antipseudomonal penicillins (without beta-lactamase inhibitors), whereas cephalosporins currently in use, for example, cefazolin, cefuroxime, ceftriaxone, and ceftazidime as well as ampicillin were unreliable.^[3]^ In contrast, this larger review suggested tetracyclines and TMP/SMX are active more frequently than found in our smaller sample.

**Table 3:** Radiographic features of pneumonia.

Radiographic appearance of pneumonia	Number of cases (n)	Percentage of total (%)
Interstitial infiltrates	15	62.50
Interstitial infiltrates and consolidation	1	4.17
Interstitial infiltrates and cavitary lesion	2	8.33
Cavitary lesion only	1	4.17
Consolidation only	1	4.17
Unspecified	4	16.67

## 4 Discussion

The hallmarks of *B bronchiseptica* infections in patients with HIV/AIDS are a respiratory syndrome, most frequently characterized as an atypical pneumonia with persistent cough, in individuals with advanced immune suppression as evidenced by substantial CD4 lymphocyte depletion. Indeed, the CD4 lymphocyte count was less than 100 cells/μL in over 80% of patients whose CD4 lymphocyte count was reported. This finding is consistent with HIV depleting CD4^+^ T lymphocyte subsets in the lung and the notion that these cells are critical for immunity against *B bronchiseptica*.^[8,16]^ This theme of advanced immune suppression is also seen in many cases of *B bronchiseptica* infection without HIV, but with clear immunosuppressive conditions such as acute leukemia, solid organ or bone marrow transplantation, as well as other chronic conditions that compromise host defenses such as alcoholism, diabetes, and renal failure.^[1,3,17,18]^ Nevertheless, immune suppression is not invariable as rare cases are reported in individuals presumed to be immunocompetent.^[3]^

Animal contact is another feature that is common, but not invariably found. Our survey found that 55% of patients with *B bronchiseptica* reported animal contact, a proportion within the range of the 48% to 68% of American households reported to own pets.^[19]^ Thus, individuals with HIV/AIDS and animal contact do not seem over-represented among those with infection and lack of animal contact should not exclude the diagnosis as a possibility. Nonetheless, a vaccine for *B bronchiseptica* is available for dogs and cats and immunosuppressed pet owners should be advised to vaccinate their pets.^[20]^

*B bronchiseptica* in patients with HIV/AIDS typically causes symptoms ranging from mild to moderate upper respiratory symptoms, for example, cough, to pneumonia, fulminant sepsis, and ARDS. Imaging findings are nonspecific, including increased interstitial markings, nodules, ground glass opacities, and cavitary nodules.^[12]^*B bronchiseptica* is typically diagnosed via culture of respiratory tract secretions and blood as a Gram negative coccobacillus that will grow on standard media such as sheep blood and chocolate agar. Some authors have suggested bacterial colony counts greater than 10^4^ CFU/mL in bronchoalveolar lavage fluid could differentiate colonization of the respiratory epithelium from infection.^[21]^ However, since *B bronchiseptica* is rarely isolated and clearly has pathogenic potential, this threshold should be used cautiously in immunocompromised patients with compatible clinical syndromes. In our clinical microbiology laboratory, *B pertussis, B parapertussis*, and *B bronchiseptica* are initially identified as a *Bordetella* group by MALDI then supplementary tests such as biochemical and growth characteristics are used to specify *B bronchiseptica*.^[15]^ There is also a risk of it being identified as *B pertussis* by multiplex molecular assay.^[22]^

Key strengths of this paper are the comprehensive nature of the review of *B bronchiseptica* infections in patients with HIV/AIDS, and the high applicability of results in this unique group of patients to those who are immunocompromised by other conditions. We also show that pet exposure is not the strong risk factor previously reported and that some antibiotics commonly used in patients with HIV/AIDS, for example, TMP/SMX, do not have reliably high activity against *B bronchiseptica.* Notable limitations include the retrospective nature of the work and that antimicrobial susceptibility testing was not standardized across all reports such that some antibiotics were tested on few strains.

Collectively this review shows that *B bronchiseptica* infection in patients with HIV/AIDS, as well as others with advanced immune suppression, largely constitutes a respiratory syndrome, most frequently characterized as an atypical pneumonia with persistent cough. It can be misidentified as *B pertussis* and should be positively identified by a combination of techniques, for example, matrix assisted laser desorption ionization time of flight or multiplex molecular assay followed by supplemental biochemical tests. Although treatable, it can lead to sepsis and death. *B bronchiseptica* is reliably susceptible to antipseudomonal penicillins combined with beta-lactamases, carbapenems, and fluoroquinolones whereas cephalosporins, including 3^rd^ and 4^th^ generation drugs are unreliable. These drugs are commonly used as empiric therapy for pneumonia in immunocompromised patients and definitive antibiotic therapy should be guided by susceptibility testing. As with other rare infections, there are no guidelines on duration of treatment. Thus, duration of antibiotics should be determined by the severity of infection, response to initial therapy, and careful follow-up. Most patients identified by this review received approximately 2 weeks of antibiotics.

## Acknowledgments

The authors thank Denise Robison MS, MT (ASCP) SM for her assistance.

## Author contributions

VG, participated in clinical care of the patient, performed the literature review, and drafted and edited the manuscript. BA and MM participated in clinical care of the patient and drafting and editing of the manuscript. DS oversaw diagnostic microbiology and edited the manuscript, DD set overall goals and objectives for the manuscript, oversaw and participated in drafting and editing of the manuscript, sought approval from the institutional IRB, and mentored VG in manuscript writing and publication. All authors have read and approved the final manuscript.

**Conceptualization:** Douglas A. Drevets.

**Data curation:** Veena Gujju, Douglas A. Drevets.

**Investigation:** Veena Gujju, Douglas A. Drevets.

**Methodology:** Douglas A. Drevets.

**Project administration:** Douglas A. Drevets.

**Resources:** Douglas A. Drevets.

**Supervision:** Miranda A. McGhee, Douglas A. Drevets.

**Validation:** Douglas A. Drevets.

**Visualization:** Douglas A. Drevets.

**Writing – original draft:** Veena Gujju, Bushra Akram, Miranda A. McGhee.

**Writing – review & editing:** Veena Gujju, Dena R. Shibib, Miranda A. McGhee, Douglas A. Drevets.

## References

[1] Goodnow RA. Biology of *Bordetella bronchiseptica*. Microbiol Rev 1980;44:722–38. 7010115 10.1128/mr.44.4.722-738.1980 PMC373201

[2] Mattoo S Cherry JD. Molecular pathogenesis, epidemiology, and clinical manifestations of respiratory infections due to *Bordetella pertussis* and other *Bordetella* subspecies. Clin Microbiol Rev 2005;18:326–82. 15831828 10.1128/CMR.18.2.326-382.2005 PMC1082800

[3] Woolfrey BF Moody JA. Human infections associated with *Bordetella bronchiseptica*. Clin Microbiol Rev 1991;4:243–55. 1889042 10.1128/cmr.4.3.243 PMC358197

[4] Egberink H Addie D Belák S. *Bordetella bronchiseptica* infection in cats. ABCD guidelines on prevention and management. J Feline Med Surg 2009;11:610–4. 19481041 10.1016/j.jfms.2009.05.010 PMC11132281

[5] Kamanova J. *Bordetella* type III secretion injectosome and effector proteins. Front Cell Infect Microbiol 2020;10:466. 33014891 10.3389/fcimb.2020.00466 PMC7498569

[6] Yuk MH Harvill ET Cotter PA Miller JF. Modulation of host immune responses, induction of apoptosis and inhibition of NF-κB activation by the *Bordetella* type III secretion system. Mol Microbiol 2000;35:991–1004. 10712682 10.1046/j.1365-2958.2000.01785.x

[7] Gueirard P Bassinet L Bonne I Prevost MC Guiso N. Ultrastructural analysis of the interactions between *Bordetella pertussis*, *Bordetella parapertussis* and *Bordetella bronchiseptica* and human tracheal epithelial cells. Microb Pathog 2005;38:41–6. 15652294 10.1016/j.micpath.2004.08.003

[8] Pilione MR Harvill ET. The *Bordetella bronchiseptica* type III secretion system inhibits gamma interferon production that is required for efficient antibody-mediated bacterial clearance. Infect Immun 2006;74:1043–9. 16428751 10.1128/IAI.74.2.1043-1049.2006 PMC1360352

[9] Skinner JA Pilione MR Shen H Harvill ET Yuk MH. *Bordetella* type III secretion modulates dendritic cell migration resulting in immunosuppression and bacterial persistence. J Immunol 2005;175:4647–52. 16177111 10.4049/jimmunol.175.7.4647

[10] Bendor L Weyrich LS Linz B. Type six secretion system of *Bordetella bronchiseptica* and adaptive immune components limit intracellular survival during infection. PLoS One 2015;10:e0140743. 26485303 10.1371/journal.pone.0140743 PMC4618060

[11] Warfel JM Beren J Merkel TJ. Airborne transmission of *Bordetella pertussis*. J Infect Dis 2012;206:902–6. 22807521 10.1093/infdis/jis443 PMC3501154

[12] Patel AK Prescott-Focht JA Kunin JR Essmyer CE Rosado-de-Christenson ML. Imaging findings in human *Bordetella bronchiseptica* pneumonia. J Thorac Imaging 2011;26:W146–9. 21263355 10.1097/RTI.0b013e31820209a1

[13] Amador C Chiner E Calpe JL de la Table O V Martinez C Pasquau F. Pneumonia due to *Bordetella bronchiseptica* in a patient with AIDS. Rev Infect Dis 1991;13:771–2. 1925302 10.1093/clinids/13.4.771

[14] Ho J Hussain S Sparagano O. Did the COVID-19 pandemic spark a public interest in pet adoption? Front Vet Sci 2021;8:444. 10.3389/fvets.2021.647308 PMC8145284 34046443

[15] Winn W Allen S Janda W. Koneman's Color Atlas and Textbook of Diagnostic Microbiology. 6th ed. New York: Lippincott Williams and Wilkins; 2006.

[16] Kolls JK. CD4(+) T-cell subsets and host defense in the lung. Immunol Rev 2013;252:156–63. 23405903 10.1111/imr.12030 PMC3576701

[17] Dlamini NR Bhamjee A Levick P Uniacke E Ismail H Smith A. Spontaneous bacterial peritonitis and pneumonia caused by *Bordetella bronchiseptica*. J Infect Dev Ctries 2012;6:588–91. 22842947 10.3855/jidc.2074

[18] Yacoub AT Katayama M Tran J Zadikany R Kandula M Greene J. *Bordetella bronchiseptica* in the immunosuppressed population – a case series and review. Mediterr J Hematol Infect Dis 2014;6:e2014031. 24804004 10.4084/MJHID.2014.031 PMC4010603

[19] Brulliard K Clemet S. How many Americans have pets? An investigation of fuzzy statistics. The Washington Post 2021.

[20] Woodard DR Cone LA Fostvedt K. *Bordetella bronchiseptica* infection in patients with AIDS. Clin Infect Dis 1995;20:193–4. 7727654 10.1093/clinids/20.1.193

[21] Sameed M Sullivan S Marciniak ET Deepak J. Chronic cough and cystic lung disease caused by *Bordetella bronchiseptica* in a patient with AIDS. BMJ Case Rep 2019;12:04. 10.1136/bcr-2018-228741 PMC6506123 30996069

[22] McNulty MC Shibib DR Steinbeck JL. Misdiagnosis of *Bordetella bronchiseptica* respiratory infection as *Bordetella pertussis* by multiplex molecular assay. Clin Infect Dis 2018;67:1919–21. 29860317 10.1093/cid/ciy469

